# Hypokalemic quadriplegia in Sjogren's syndrome: A case report

**DOI:** 10.1002/ccr3.9227

**Published:** 2024-07-31

**Authors:** Mahsa Mehdipour Dalivand, Rezvan Abdolazimi, Majid Alikhani

**Affiliations:** ^1^ Guilan Rheumatology Research Center, Department of Rheumatology, Razi Hospital, School of Medicine Guilan University of Medical Sciences Rasht Iran; ^2^ Rheumatology Research Center Tehran University of Medical Sciences Rasht Iran

**Keywords:** autoimmune diseases, hypokalemia, paralysis, renal tubular acidosis, Sjogren's syndrome

## Abstract

**Key Clinical Message:**

In managing Sjogren's syndrome, a thorough patient history, proper lab tests, and imaging are crucial. Clinicians should prioritize checking electrolyte levels in cases of muscle weakness, as early detection of hypokalemia can prevent severe complications. Proactive monitoring can avert renal tubular acidosis and improve patient outcomes.

**Abstract:**

Distal renal tubular acidosis (dRTA) occurs in approximately one‐third of patients with Sjogren's syndrome, a systemic autoimmune disorder characterized by lymphocytic infiltration of exocrine glands, leading to dryness of mucous membranes. Hypokalemic paralysis, a well‐documented but rare complication of dRTA, typically manifests as symmetric proximal muscle weakness of the extremities. We present the case of a 38‐year‐old woman with a history of Sjogren's syndrome diagnosed 3 years prior, who ceased her medication without medical supervision. She presented with quadriplegia, initially beginning unilaterally. This particular presentation is seldom documented in the literature. Laboratory investigations revealed hypokalemia and normal anion gap metabolic acidosis, consistent with dRTA‐induced hypokalemic paralysis. Intravenous potassium chloride was administered, resulting in complete recovery of muscle strength. Hypokalemic paralysis associated with dRTA is typically reversible; however, delays in diagnosis and treatment can lead to life‐threatening complications such as respiratory failure and arrhythmias. Therefore, clinicians should maintain a high index of suspicion for this condition in patients presenting with muscle weakness. Prompt and precise history takingand screening, and initiating appropriate management to prevent adverse outcomes.

## 1 INTRODUCTION

Sjogren's syndrome is a systemic autoimmune disease primarily affecting the exocrine glands, with additional extra‐glandular manifestations such as renal involvement.^1^ Tubulointerstitial nephritis represents the principal renal complication, with distal renal tubular acidosis (d‐RTA) occurring in only 4.3%–9% of Sjogren's patients.^2^ Typically affecting middle‐aged women, only two‐thirds of Sjogren patients with RTA develop symptoms.^3^ Acute hypokalemic paralysis is an uncommon yet potentially life‐threatening cause of acute weakness, attributable to various etiologies including genetic, endocrine, gastrointestinal, and renal factors. d‐RTA is a major contributor to acquired hypokalemic paralysis.^4^, ^5^ A classic presentation of hypokalemic paralysis entails the sudden onset of ascending, symmetrical paralysis of proximal muscles, accompanied by diminished deep tendon reflexes and no alteration in the level of consciousness.^4^ However, instances of hypokalemic paralysis initiating with unilateral, asymmetrical weakness of limbs, as observed in the case we aim to discuss here, have been rarely documented.

## 2 CASE PRESENTATION

A 38‐year‐old woman presented to the emergency department with weakness and progressive extreme muscle weakness in her limbs. Six months prior to admission, she discontinued her medications without medical advice, leading to a gradual recurrence of symptoms. Dry mouth and eye symptoms reappeared 3 months ago, followed by polyarthralgia for which she self‐medicated with NSAIDs intermittently. Three days before admission, she experienced weakness starting in the proximal and distal regions of her left upper limb, progressing to involve her right side, ultimately resulting in an inability to walk or perform daily activities, prompting her emergency presentation.

She had been diagnosed with Sjogren's syndrome 3 years earlier, confirmed by xerophthalmia on Schirmer test, along with symptoms of dry mouth, polyarthritis, elevated erythrocyte sedimentation rate (ESR), anemia, proteinuria (sub‐nephrotic range, 500–600 mg/day), and positive serology for rheumatoid factor (RF), antinuclear antibody (FANA), anti‐RO, and anti‐LA. The diagnosis was further confirmed by lower lip (labial glands) biopsy. She had been successfully managed with 400 mg hydroxychloroquine, 10 mg prednisolone, and 75 mg azathioprine, resulting in resolution of her symptoms and improvement in laboratory parameters including ESR, proteinuria, and hemoglobin.

She denied symptoms such as dysphagia, blurred vision, urinary incontinence, skin lesions, muscle pain, or paresthesia. Over the past 6 months, her only medication had been amitriptyline, with occasional use of naproxen 500 mg three to four times weekly for joint pain. She had no history of allergies, substance abuse, or alcohol consumption.

### 2.1 Investigations and differential diagnosis

Clinical examination revealed a middle‐aged woman with normal BMI and general appearance, without signs of ptosis. Signs of arthritis were noted in her wrists. Vital signs were stable, and she was afebrile. Her neurological examination revealed normal mental status, cranial nerve function including pupillary response, and no sensory deficits were noted. However, her gait was disrupted due to muscle weakness attributed to hypokalemia. Muscle strength in her limbs was graded at 2/5 distally and proximally in both lower limbs and 3/5 in the distal and proximal regions of her upper limbs. Deep tendon reflexes were 1+ in her biceps, brachioradialis, and quadriceps, and 0+ in her ankles, with normal Babinski and sensory responses bilaterally.

Laboratory investigations for our patient are demonstrated in Table 1. Abdominopelvic ultrasound showed no evidence of nephrocalcinosis. Imaging studies of the central nervous system, including computed tomography scan and magnetic resonance imaging of the brain and spinal cord, ruled out central nervous system pathology. Electromyogram and nerve conduction velocity studies were inconclusive for peripheral nervous system involvement.

**TABLE 1:** Laboratory findings of a 38 year old female patient with Sjogren disease.

Test	Value	Normal range
White blood cell count (WBC)	5220	4500–12,500
Hemoglobin (Hb)	12.9 g/dL	12–16 g/dL
Platelet count	184,000/μL	150,000‐450,000/μL
Mean corpuscular volume (MCV)	88 fL	80–98 fL
Blood urea nitrogen (BUN)	17 mg/dL	7–20 mg/dL
Creatinine (Cr)	1.5 mg/dL	0.5–1.3 mg/dL
Sodium (Na)	140 mmol/L	135–145 mmol/L
Potassium (K)	2.2 mmol/L	3.5–5.5 mmol/L
Calcium (Ca)	8.9 mg/dL	8.5–10.5 mg/dL
Albumin (Alb)	4.6 g/dL	3.4–5.4 g/dL
Magnesium (Mg)	2.4 mg/dL	1.8–2.6 mg/dL
pH	7.29	7.35–7.45
Partial pressure of carbon dioxide (PCO^2^)	25 mmHg	35–45 mmHg
Bicarbonate (HCO^3^)	9.4 mmol/L	24–32 mmol/L
Chloride (Cl)	121 mmol/L	96–106 mmol/L
Aspartate aminotransferase (AST)	18 U/L	<31 U/L
Alanine aminotransferase (ALT)	15 U/L	<31 U/L
Alkaline phosphatase (ALKP)	161 U/L	80–300 U/L
Erythrocyte sedimentation rate (ESR)	110 mm/h	0–25 mm/h
Thyroid‐stimulating hormone (TSH)	8 mIU/L	0.25–5 mIU/L
Thyroxine (T4)	9.6 μg/dL	5.1–14.1 μg/dL
Triiodothyronine (T3)	1.7 ng/mL	1.3–3.1 ng/mL
Creatine phosphokinase (CPK)	42 U/L	10–120 U/L
Aldolase	2.5 U/L	<7.7 U/L
25‐Hydroxy‐vitamin D	23.5 ng/mL	30–100 ng/mL
HIV	Negative	Negative
HBsAg	Negative	Negative
Anti HCV	Negative	Negative
Urine analysis	Specific gravity: 1015, urine pH: 6.5, protein: 1+, blood: negative; 24 h urine: vol = 1900, protein = 608, creatin = 455	‐

Inflammatory myopathies and hypothyroid‐induced myopathy were considered but were not supported by laboratory findings. Although her thyroid‐stimulating hormone (TSH) level was elevated, her free thyroxine (T4) and triiodothyronine (T3) levels were within normal limits and unlikely to explain her symptoms. She was diagnosed with hypokalemic‐induced paralysis due to d‐RTA, possibly secondary to Sjogren's syndrome or exacerbated by NSAID use, albeit at subtherapeutic doses.

### 2.2 Treatment, outcome and follow‐up

Treatment commenced with intravenous potassium chloride, in which initially we administered 20 mEq of KCl orally three times a day, along with 20 mEq of intravenous potassium twice a day. The patient's weakness began to resolve following potassium therapy, with significant improvement observed after the first day. By the second day, her muscle strength was 4/5, and on the third day, she was able to walk without assistance and regained full strength in all limbs. Sjogren's syndrome management was reinstated with prednisolone, azathioprine, and hydroxychloroquine, leading to gradual resolution of joint pain, dry eye, and dry mouth symptoms. She was discharged after 6 days from her admission with potassium citrate tablets, along with 30 mg prednisolone, 50 mg azathioprine, and 400 mg hydroxychloroquine.

## 3 DISCUSSION

Sjogren's syndrome is a systemic chronic inflammatory disorder characterized by lymphocytic infiltrations in exocrine organs.^6^ Alongside these glandular symptoms, patients may experience extra‐glandular manifestations such as arthralgia, arthritis, gastrointestinal and pulmonary issues, anemia, lymphadenopathy, and lymphoma, with renal involvement also documented in around 5% of SS patients.^2^, ^7^ Tubulointerstitial nephropathy (TIN) represents the most common form of nephropathy in Sjogren's syndrome, characterized by plasma cell infiltration akin to that observed in salivary glands. Electrolyte disturbances, including d‐RTA, diabetes insipidus, Gitelman syndrome, or Fanconi syndrome, may also manifest.^8^

The precise mechanism underlying d‐RTA in Sjogren's syndrome remains incompletely elucidated. However, a possible mechanism involves inhibition of carbonic anhydrase 2 by high‐titer autoantibodies, leading to defective H+ secretion.^9^ Hypokalemia represents the most common electrolyte abnormality in d‐RTA, with causes including decreased distal tubular sodium delivery, secondary hyperaldosteronism, defective H‐KATPase, and bicarbonaturia.^10^ Although hypokalemia is common in RTA, severe hypokalemia resembling periodic paralysis is rare, as evidenced by previous case reports such as that by Goroshi et al., where 13 cases of hypokalemic paralysis were ultimately diagnosed as Sjogren's syndrome, predominantly affecting females with a mean age similar to our case.^11^ Given that she also had no history of familial Hypokalemic periodic paralysis, this diagnosis was also ruled out.

Unilateral presentation of hypokalemic paralysis is exceptionally rare, as demonstrated by reports from Liu et al. and Lajeunesse et al., detailing unilateral cases in elderly patients.^12^, ^13^ In our patient, symptoms initiated unilaterally on the left side before progressing to involve other extremities, culminating in quadriplegia.

Management of d‐RTA involves correcting potassium levels prior to initiating alkali therapy, as the latter may exacerbate hypokalemia by promoting potassium shift into cells and bicarbonaturia.^14^ There is still no consensus regarding the utilization of immunomodulatory therapy in RTA. While RTA itself does not typically warrant immunomodulatory therapy in Sjogren's syndrome, despite its status as an extra‐glandular manifestation,^11^ previous reports have indicated effective treatment outcomes for RTA in SS using immunosuppressive medications such as Cyclophosphamide.^15^ In our case, full recovery was achieved without the use of immunomodulatory medication.

Renal prognosis in Sjogren's syndrome with TIN is generally favorable, although the risk of chronic kidney disease remains elevated. Therefore, appropriate screening measures are essential for early detection of renal complications.^8^

## 4 CONCLUSION

It is crucial to take a precise patient history and employ appropriate laboratory tests and imaging in the management of SS patients. In clinical practice, step‐by‐step evaluation is essential; for instance, when managing patients presenting with abnormal muscle forces, electrolyte levels should be the initial consideration. Although RTA is a rare complication of Sjogren's syndrome, clinicians should remain vigilant for its presence in Sjogren's patients presenting with hypokalemia. Regular monitoring of serum potassium levels, venous blood gases (VBG), bicarbonate levels, and urine tests during follow‐up visits is advisable for early detection and prevention of RTA in Sjogren's syndrome. While specific guidelines for RTA prevention in Sjogren's syndrome are currently lacking, proactive monitoring can help in managing electrolyte imbalances effectively. Hypokalemic paralysis is typically reversible, yet its associated hypokalemia can precipitate fatal complications such as respiratory failure and arrhythmias. Thus, maintaining a high index of suspicion for hypokalemia as a differential diagnosis in cases of weakness, regardless of the presentation type, is imperative for prompt intervention and optimal patient outcomes.

## AUTHOR CONTRIBUTIONS

**Mahsa Mehdipour Dalivand:** Conceptualization; writing – original draft. **Rezvan Abdolazimi:** Investigation; supervision. **Majid Alikhani:** Conceptualization; resources; supervision; validation.

## FUNDING INFORMATION

No financial support was received for this report.

## CONFLICT OF INTEREST STATEMENT

The authors declare that they have no competing interests.

## ETHICS STATEMENT

The present study was approved by the Medical Ethics Committee of the University. The patients provided both oral and written informed consents for the publishing of this report and any accompanying images. All identifying information has been removed to preserve confidentiality.

## CONSENT

Written informed consent was obtained from the patient to publish this report in accordance with the journal's patient consent policy. A copy of the written consent is available for review by the corresponding author.

## Data Availability

All data regarding this case report has been reported in the manuscript. Please contact the corresponding author in case of requiring any further information.
